# Identifying potential drivers of distribution patterns of invasive *Corbicula fluminea* relative to native freshwater mussels (Unionidae) across spatial scales

**DOI:** 10.1002/ece3.8737

**Published:** 2022-03-18

**Authors:** Taylor E. Kelley, Garrett W. Hopper, Irene Sánchez González, Jamie R. Bucholz, Carla L. Atkinson

**Affiliations:** ^1^ 8063 Department of Biological Sciences University of Alabama Tuscaloosa Alabama USA

**Keywords:** filter‐feeders, invasive species, niche overlap, niche partitioning, positive associations, rivers, species interactions

## Abstract

This study aimed to identify the importance of ecological factors to distribution patterns of the invasive Clam (*Corbicula fluminea*) relative to native mussels (family: Unionidae) across seven rivers within the Mobile and Tennessee basins, Southeast United States. We quantitatively surveyed dense, diverse native mussel aggregations across 20 river reaches and estimated mussel density, biomass, and species richness along with density of invasive *C*. *fluminea* (hereafter *Corbicula*). We measured substrate particle size, velocity, and depth in quadrats where animals were collected. Additionally, we characterized reach scale environmental parameters including seston quantity and quality (% Carbon, % Nitrogen, % Phosphorous), water chemistry (ammonium [${\mathrm{NH}}_{4}^{+}$], soluble reactive phosphorous [SRP]), and watershed area and land cover. Using model selection, logistic regression, and multivariate analysis, we characterized habitat features and their association to invasive *Corbicula* within mussel beds. We found that *Corbicula* were more likely to occur and more abundant in quadrats with greater mussel biomass, larger substrate size, faster water velocity, and shallower water depth. At the reach scale, *Corbicula* densities increased where particle sizes were larger. Mussel richness, density, and biomass increased with watershed area. Water column ${\mathrm{NH}}_{4}^{+}$ increased at reaches with more urban land cover. No land cover variables influenced *Corbicula* populations or mussel communities. The strong overlapping distribution of *Corbicula* and mussels support the hypothesis that *Corbicula* are not necessarily limited by habitat factors and may be passengers of change in rivers where mussels have declined due to habitat degradation. Whether *Corbicula* is facilitated by mussels or negatively interacts with mussels in these systems remains to be seen. Focused experiments that manipulate patch scale variables would improve our understanding of the role of species interactions (e.g., competition, predation, facilitation) or physical habitat factors in influencing spatial overlap between *Corbicula* and native mussels.

## Graphical abstract

Identifying environmental factors associated with the distribution of invasive species relative to native species is fundamental to conservation and management. Our quantitative surveys of dense, diverse native mussel beds in rivers across a broad geographic area showed that invasive *Corbicula fluminea* were more likely to occur and more abundant where mussels are. The strong positive association between invasive and native bivalves support the hypothesis that *C*. *fluminea* are not necessarily limited by habitat factors and may be passengers of change in rivers where mussels have declined due to habitat degradation.

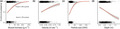

## 1 INTRODUCTION

Invasive species are a leading threat to native biodiversity (Clavero & García‐Berthou, 2005; Wilcove et al., 1998). Invasive species often exert negative pressures on native species through predation and competition (Davis, 2003) which can contribute to their successful establishment (Gamradt & Kats, 1996; Simberloff et al., 2020). When invasive species are functionally similar to native species, their competitive effects can be particularly harmful because of their overlapping resource requirements (Booth et al., 2003; David et al., 2017). However, theory predicts that competitive exclusion will limit the coexistence of functionally similar species (Levin, 1970; Macarthur & Levins, 1967); thus, native communities may also suppress invasion by functionally similar species due to limiting similarity (Fargione et al., 2003; Tilman, 2004). In either case, non‐overlapping distributions between invasive and native species have been used to support speculations of competitive exclusion, suppression, or biotic homogenization (Fargione & Tilman, 2005; Padial et al., 2020). However, the Anthropocene is characterized by significant declines in native biodiversity, making it equally possible that patterns of overlap between native and invasive species are driven by functionally similar species invading habitats once occupied by their native counterparts (MacDougall & Turkington, 2005; Strayer et al., 1999). Therefore, characterizing the spatial overlap of invasive species relative to native communities and the habitat that control invasive species populations is essential for designing appropriate control measures for invasive species and recovery plans for native species (Pergl et al., 2020).

Freshwater ecosystems are particularly vulnerable to species introductions and extinctions because of their high degree of isolation and endemism (Reid et al., 2019). Consequently, invasive species are a prominent component of contemporary freshwater ecosystems and are implicated in populations declines or extirpations of many species (Strayer et al., 1999; Strayer & Dudgeon, 2010). In particular, invasive bivalves such as zebra mussels (*Dreissenia polymorpha*) and the invasive Clam (*Corbicula fluminea*) continue to spread and negatively affect freshwater ecosystems worldwide (Sousa et al., 2014; Strayer et al., 2014). This can be problematic for effected ecosystems with diverse and abundant communities of functionally similar native bivalves because of apparent similarities in niche requirements (Atkinson et al., 2010). Once established, invasive bivalves can dominate communities and physically alter benthic habitats (Ilarri et al., 2015; Sousa et al., 2009). Thus, quantifying biotic and abiotic controls over their invasive range is a fundamental first step in identifying potential effects on native communities.

Freshwater mussels (Bivalvia: Unionidae) are long‐lived, benthic, filter‐feeding bivalves (Haag, 2012). Mussels are common in eastern North American streams where they are patchily distributed at multiple spatial scales. Mussel life‐histories are unique, such that adults are sedentary and release larvae that are ectoparasites on fish (Barnhart et al., 2008). Therefore, mussel distributions at regional scales are partially influenced by fish host populations (Schwalb et al., 2013; Vaughn & Taylor, 2000), but once settled persistence is largely governed by environmental factors (Sansom et al., 2018). Mussels often occur as dense, species‐rich aggregations called mussel beds where mussels are 10–100× denser than in areas outside of beds (Strayer, 2008). Further, mussel densities within these beds can vary with stream size (Atkinson et al., 2012; Hopper et al., 2018). Mussel beds exist in river channels that experience significant sediment mobility, but beds can persist in the same stream sites and have similar abundance and species composition for decades (Sansom et al., 2018). Mussels are also heterogeneous within beds, with individual mussels aggregating in dense patches separated by areas with few or no mussels (Atkinson & Forshay, 2022; Vaughn & Spooner, 2006b). Mussels are crucial for ecosystem function as they filter particles from the water column and excrete and egest nutrients that are important to green (Vaughn et al., 2008) and brown food webs (Atkinson et al., 2021; Hopper et al., 2021). Unfortunately, mussels account for nearly half of imperiled species in freshwater ecosystems (Lopes‐Lima et al., 2018) and their declines have been influenced by habitat destruction, disease, climate change, and invasive species (Böhm et al., 2020). In North American rivers, of the more than 300 species of mussels, 74% are considered imperiled, and at least 35 are considered extinct (Patterson et al., 2018). Although freshwater mussels are effected by several factors involving habitat degradation and modification, and sometimes unknown reasons (Haag, 2019), the increase of invasive populations of functionally similar bivalves of the genus *Corbicula* further threatens freshwater mussel populations (Haag et al., 2020).

Bivalves of the genus *Corbicula* are native to Southeast Asia, the Middle East, Australia, and Africa (Araujo et al., 1993). Specifically, *Corbicula fluminea* (hereafter *Corbicula*) is distributed across all continents except Antarctica and was introduced in the United States on the west coast in the early 1900s (Crespo et al., 2015). Human‐mediated dispersal for various reasons (e.g., food, fish bait, aquarium releases) has promoted *Corbicula* introduction and establishment in new ecosystems (Ferreira‐Rodríguez et al., 2019; Strayer, 2010). Rapid growth, early sexual maturity, short lifespan, and high fecundity (Sousa et al., 2008) are traits that have aided in its successful establishment. *Corbicula* colonization can alter biogeochemical cycles controlled by native mussels, reduce phytoplankton abundance, alter benthic communities (Atkinson et al., 2011; Hakenkamp et al., 2001; Novais et al., 2017), or directly compete for habitat with native filter‐feeders (Ferreira‐Rodríguez et al., 2018; Ferreira‐Rodríguez & Pardo, 2017). Experimental evidence supports these observations, demonstrating that growth, physiological condition, and behavior of a native mussel (*Unio delphinus*) is reduced under increased densities of co‐occurring *Corbicula* (Ferreira‐Rodríguez et al., 2018), suggesting the displacement of native mussels to less favorable habitats by *Corbicula* may drive mussel declines. Although research conducted on *Corbicula* shows negative effects on native communities, dense populations of *Corbicula* often co‐occur with native freshwater mussels (Bódis et al., 2011; Modesto et al., 2019; Vaughn & Spooner, 2006a). Because the effects of habitat loss and disturbance also weigh heavily in many invaded systems, it is conceivable that invasive species success may be less attributable to competitive ability than expected. Indeed, the native mussel communities may not interact strongly with *Corbicula* in such a way that causes change to mussel communities, but rather *Corbicula* may be passengers of more fundamental environmental change that is most limiting to native mussels (MacDougall & Turkington, 2005; Strayer et al., 1999, 2004). Thus, identifying habitat characteristics that support *Corbicula* in habitats where mussels are abundant is key to understanding the causes and consequences of spatial overlap between native mussels and *Corbicula*.

Physical habitat variables and species interactions can influence species distributions differently depending on the spatial scale. Indeed, biotic effects are more often quantifiable and observed at fine spatial scales where species interact, whereas physical environmental variables are often more important at regional scales (Bengtsson, 1989). Here, we tested whether *Corbicula* populations were associated with physical habitat variables and native mussels across two spatial scales to identify characteristics associated with patterns of *Corbicula* distribution in native mussel beds across a wide range of physiography. Our specific objectives were to address: (1) how *Corbicula* occurrence and density vary with mussel species richness, density, and biomass at the patch and reach scale; and (2) how stream benthic habitat characteristics such as depth, particle size, and water velocity influence *Corbicula* occurrence and density at the patch and reach scale. We hypothesized that in patches and reaches where mussels are more abundant or had greater species richness *Corbicula* would be absent or at low densities due to lack of space, physical displacement by burrowing activities, and potentially reduced patch scale food resources due to competitive effects. Alternatively, *Corbicula* may invade habitats where mussel communities are already in decline because of anthropogenic activities, such as land use practices that increase non‐point source nutrient loading (MacDougall & Turkington, 2005). In this case, we expected *Corbicula* occurrence and density to be greatest in patches and reaches with more mussel species and greater densities. Next, we hypothesized that *Corbicula* would be associated with physical habitat characteristics where mussels occur due to functional similarity. Overall, our findings provide a more nuanced view of the abiotic factors underlying *Corbicula* occurrence and abundance and the potential spatial overlap with native bivalve communities.

## 2 MATERIALS AND METHODS

### 2.1 Study region

**FIGURE 1:**
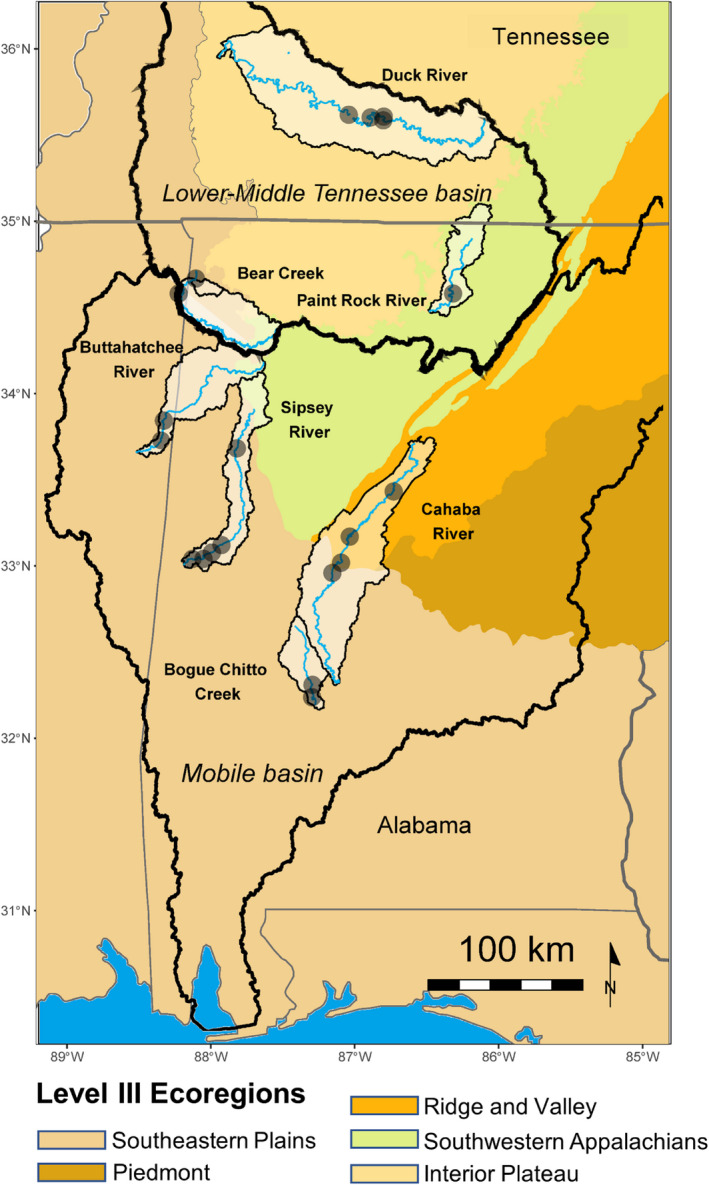
Map of the study area with defined Level III ecoregions. Sample sites are the black points. Focal watersheds are highlighted within major drainage basins

North American rivers contain the greatest known diversity of freshwater mussels (~360 species), and the Mobile and Tennessee River Basins represent ~60% of that diversity (Williams et al., 2008). Various human activities have degraded rivers in this region, and ~95% of U.S. federally protected mussels can be found in this region. While *Corbicula* is suspected to harm mussels and was established in these rivers more than 50 years ago, invasion timing and quantitative population estimates are not widely available (Benson & Williams, 2021). We selected three rivers in the Tennessee Basin and four in the Mobile Basin with variable mussel densities and species composition to evaluate how *Corbicula* populations are distributed across ecological gradients (Figure 1). The Paint Rock River, Bear Creek, and Duck River are tributaries to the Tennessee River and support high mussel diversity (Paint Rock 58 species, Bear Creek 34 species, Duck 68 species) and vary in the watershed area (Paint Rock 1191 km^2^, Bear Creek 2450 km^2^, Duck 8100 km^2^). The Sipsey (watershed area 2044 km^2^) and Buttahatchee River (watershed area 2252 km^2^) occur in the Mobile basin as tributaries to the Tombigbee River and maintain historical mussel communities (Sipsey 42 species, Buttahatchee 43 species). The Cahaba River (watershed area 3009 km^2^) and Bogue Chitto Creek (watershed area 937 km^2^) are tributaries to the Alabama River before meeting the Mobile River in southwestern Alabama. Both have been effected negatively (e.g., recent droughts, habitat degradation, and invasive species), but historically had diverse mussel communities with 50 species in the Cahaba (Onorato et al., 2000) and 20 species in Bogue Chitto Creek (Sánchez González et al., 2021).

### 2.2 Bivalve sampling

We sampled quadrats (patches) nested within sites (mussel bed reaches) to make comparisons across rivers and two spatial scales. We intentionally surveyed sites encompassing a wide range of mussel abundance and richness to examine the range of *Corbicula* densities within areas where unionids occur and to quantify the effects of variation in mussel abundance on *Corbicula*. We quantified *Corbicula* and mussel densities at five sites in the Sipsey River, two in Bear Creek, one in the Paint Rock River, two in the Buttahatchee River and two in Bogue Chitto Creek in 2018 and 2019, and four sites in the Duck River and Cahaba River during 2020 all at base‐flow (Figure 1). We excavated 0.25 m^2^ quadrats to 15 cm deep traversing the river's width every 2.5 m at four random transects every 20 m along the entire reach (as in Hopper et al., 2021). Total reach length varied (range 40–150 m). We measured the length of all mussels and a minimum of 100 *Corbicula* found in quadrats along the longest shell axis (mm) at each site. Length‐mass regressions were used to estimate soft tissue dry mass for mussels (STDM (g); Atkinson et al., 2020), and reach level areal biomass was based on averages of the quadrat estimates (g m^−2^).

### 2.3 Patch‐scale environmental factors

We measured depth (m) and velocity (m s^−1^) at each quadrat using a Hach FH950 flow meter (Hach Company, Loveland, CO). We calculated D_50_ from pebble counts (Wolman, 1954) within quadrats (*n* = 10 pebbles/quadrat) to describe substrate heterogeneity across spatial scales (patch and reach). Substrate data was not collected at one site in the Sipsey River (Sipsey 2) and is therefore excluded from the analysis of abiotic drivers.

### 2.4 Reach‐scale environmental factors

To address watershed characteristics that might mediate *Corbicula* abundances at mussel aggregations, we determined the percentage of watershed land cover (e.g., agriculture, forest) for each site using data from the National Land Cover Database (NLCD) clipped to the watershed area upstream of each site. During low flow conditions in 2019 and 2020, we collected triplicate filtered water samples (ashed, pre‐weighed GF/F; 0.7‐µm pore size; Millipore) to quantify variation in background nutrient concentrations. Water samples were kept in a cooler with ice until arrival to the lab, where samples were frozen at −20°C until nutrient analysis. We used a Seal AQ300 discrete analyzer (Seal Analytical) to analyze SRP (hereafter P) using the colorimetric method (Murphy & Riley, 1962) and ${\mathrm{NH}}_{4}^{+}$ (hereafter N) using the phenol method. We measured pH once using a YSI professional plus multiparameter meter (YSI Inc. Yellow Springs, Ohio, USA). We also measured seston quality and quantity at each site in 2019 and 2020. For seston quantity, we filtered one liter of stream water (*n* = 3/site/year) on ashed, pre‐weighed filters (GF/F; 0.7‐µm pore size; Millipore). The filtered materials were taken to the lab, dried at 50 °C in a convection oven (VWR 414005–106) for 48 h, weighed for dry mass (mg), followed by combustion at 500°C for two hours, and reweighed for ash‐free dry mass (AFDM). For seston quality, we filtered 1–3 L of water on ashed filters (GF/F; 0.7‐µm pore size; Millipore) for the determination of %C, %N, and %P. We subsampled our dried filters and measured %C and %N using a Carlo Erba CHNS‐O EA1108‐Elemental Analyzer (Isomass Scientific, Calgary, Alberta, Canada). For percent P, subsamples were weighed, combusted at 500°C for two hours, and analyzed with HCl digestion followed by soluble reactive P analysis. Lastly, we calculated D_50_ from pebble counts (n=100) to describe substrate heterogeneity at each site (Wolman, 1954).

### 2.5 Statistical analysis

Of the 1775 quadrats sampled across rivers, 154 had incomplete abiotic data (~8%). We used only quadrats with complete data fields for modeling with both abiotic and biotic factors. Sample sizes for these models across rivers were as follows: Bear Creek (*n* = 164), Bogue Chitto Creek (*n* = 76), Buttahatchee River (*n* = 225), Cahaba River (*n* = 292), Duck River (*n *= 329), Paint Rock River (*n* = 56), and Sipsey River (*n* = 479).

To test whether species interactions, habitat characteristics or the combined influence of habitat and species interactions most strongly regulate *Corbicula* abundance at the patch scale, we fit generalized linear mixed models (GLMM; *glmer* function) in R (R Core Team, 2021; Zuur, 2019) and compared them using AIC (Burnham & Anderson, 2009). We exclusively used mussel biomass (STDM m^−2^) to test the hypothesis related to biotic interactions because variance inflation factors (VIF) for mussel biomass, density, and mussel species richness were >5. Biomass incorporates aspects of both density and mussel species richness as species‐specific length‐mass models were used to estimate biomass (Atkinson et al., 2020). However, we present correlations between mussel biomass, density, and richness for completeness (see below). The first set of models evaluated the probability of *Corbicula* occurrence (i.e., detection/non‐detection) in quadrats as a function of mussel biomass; *Corbicula* occurrence in quadrats as a function of substrate particle size, velocity, and depth; and *Corbicula* occurrence as a function of mussel biomass and abiotic factors using a binomial distribution (link = logit). Following model selection, we quantitatively tested the effects of each predictor by running separate GLMMs and visualized them using scatter plots. Next, we constructed a set of models that included *Corbicula* density (individuals m^−2^) as a function of mussel biomass; *Corbicula* density as a function of substrate particle size, velocity, and depth; *Corbicula* density as a function of mussel biomass and abiotic factors. We also fit null models for comparison. *Corbicula* density was square‐root transformed in each model to better conform to the assumption of normality and heterogeneity. River was treated as a random effect in all models. We used AIC (function *aictab*; package AICcmodavg) to determine the best‐supported model (Burnham & Anderson, 2009). Variance described by the random effect was considered as the difference between marginal R² and conditional *R* ^2^ (MuMIn Bartoń, 2019; Nakagawa & Schielzeth, 2013).

### 2.6 Multivariate analysis of general patterns

Last, we examined combined abiotic and biotic drivers to determine the spatial overlap of invasive and native species. We used principal components analysis (PCA) to visualize scaled abiotic and biotic variable relationships for quadrats among sites using the function *prcomp*. We calculated 95% confidence ellipses to show quadrats “typical” of each river using the function *stat_conf_ellipse* from the package ggpubr (Kassambara, 2020; Wickham, 2011). We used *adonis* to perform permutational multivariate analysis of variance (PERMANOVA, 999 permutations (Oksanen et al., 2019), to test for differences in quadrat scale variables visualized in the PCA and *betadisper* to test for heterogeneity of variance (Anderson, 2006; Oksanen et al., 2019). In addition, we calculated and visualized a correlation matrix using *ggcorplot* in the package corrplot (Kassambara, 2019; Wickham, 2011) to assess global relationships among variables in the quadrat level GLMM analysis. We evaluated relationships between variables measured at the reach scale by calculating and plotting an additional correlation matrix.

## 3 RESULTS

### 3.1 Biotic variables

**FIGURE 2:**
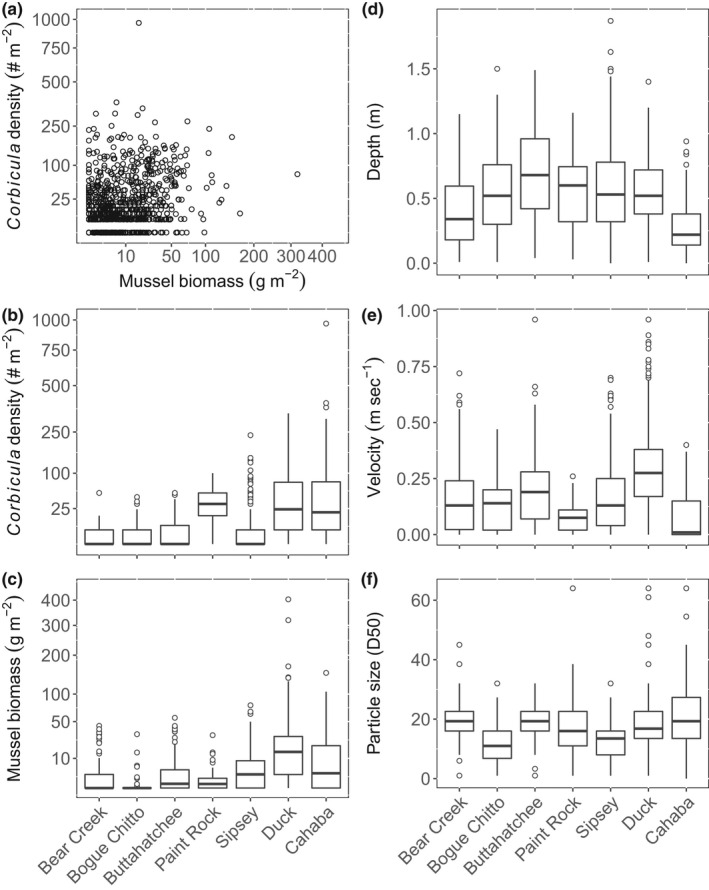
Scatter plot of *Corbicula* density and mussel biomass with square‐root transformed axes (a). Box plots showing variation in substrate particle size (d), velocity (e), Depth (f), Corbicula density (b), and mussel biomass (c) measured in quadrats across seven rivers in the southeastern USA. Boxes cover the first through third quartile of the data; horizontal black line in each box is the median. One data point representing the greatest *Corbicula* density (2088 individuals m^−2^) is excluded from panels a and b

We collected a total of 12,411 *Corbicula* and 3892 mussels from 1775 quadrats. Total mussel species richness ranged from 0 to 12 in quadrats and 4 to 32 for reaches. *Corbicula* densities ranged from 0 to ~2000 individuals m^−2^ in quadrats (Figure 2a), and mean densities for reaches ranged from 1.70 to 131.60 individuals m^−2^ (Figure 2b). Mussel densities in quadrats ranged from 0 to 148 individuals m^−2^ (Figure 2a) and at the reach scale ranged from 0.50 to 23.86 individuals m^−2^ (Figure 2c). Mussel biomass ranged from 0 to 403 g STDM m^−2^ in quadrats, and mean mussel biomass for reaches ranged from 0.46 to 40.24 g STDM m^−2^ (Appendix S1).

### 3.2 Environmental variables

**FIGURE 3:**
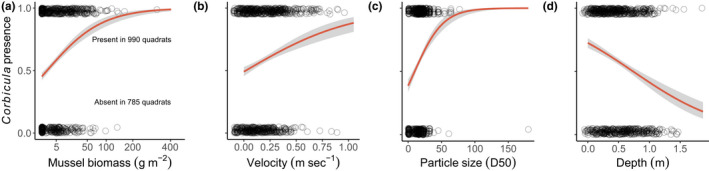
Corbicula probability of occurrence increases in quadrats with more mussel biomass (a), faster velocity (b), and larger particle sizes (c), but decreases in deeper quadrats. Points represent quadrats and are vertically jittered to avoid complete overlap. *X* axis in (a) is square‐root transformed

Quadrats varied in depth from 0 to 1.87 m (Figure 2d). Quadrats with a zero depth were typically wetted, but at the edge of the river and made up ~1% of samples. Water velocity measured in quadrats ranged from 0 to 1.05 m s^−1^ (Figure 2e). Substrate particle sizes ranged from <2 to 180 mm (Figure 2f) and calculated D_50_ for quadrats was between 1 and 65 mm (Figure 3). Bedrock and large wood represented the dominate substrate in some quadrats, but no *Corbicula* or mussels were collected from those quadrats.

Watershed area for reaches ranged 564–3119 km^2^, with agriculture land cover comprising 16.9–1453 km^2^ and developed land being 0.5–373 km^2^ (Appendix S2). Nutrient concentrations were also variable with ${\mathrm{NH}}_{4}^{+}$ concentrations from 5.73 to 28.41 µg L^−1^ and SRP from 7.04 to 112.29 µg L^−1^. The sites had pH values ranging from 6.08 to 7.96. Seston AFDM varied from 1.75 to 20.62 mg L^−1^. Seston % C ranged from 2.19 to 10.01, while seston % N ranged from 0.24 to 1.32, and seston % P varied from 0.07 to 0.91. D_50_ summarized for reach was between 8 and 16.

### 3.3 Ecological drivers of spatial overlap between Corbicula and mussels

*Corbicula* was present at all sites and was detected in 55% of sampled quadrats (Figure 3). Each hypothesis‐driven model explaining the probability of *Corbicula* occurrence in quadrats had considerably lower AIC values than the null model (Table 1), indicating each was an improvement over the null model. The model including only mussel biomass performed worst, followed by the model containing abiotic factors. The model with both abiotic factors and mussel biomass explained the most variance and performed the best (AIC = 1672.5) even with penalization for having the most variables (Table 1). All terms included in the best model were strong predictors of *Corbicula* probability of occurrence. This result indicates that *Corbicula* probability of occurrence increased in shallower quadrats with more mussel biomass, relatively larger substrate particle sizes, and faster velocities (Table 1; Figure 3). Variance attributed to the random effect of river was strong in all models (*R* ^2^ marginal – *R* ^2^ Conditional), suggesting unmeasured factors associated with ecological gradients across rivers influence model outcomes at the patch scale.

**TABLE 1:** Generalized linear models fit for each hypothesis regarding *Corbicula* presence or density as the response variable including river as a random effect

Model	AIC	Variable	χ^2^	Estimate	*p*‐Value	*R* ^2^ marginal	*R* ^2^ conditional
*Corbicula* presence							
Null	1788.4	–	–	0.49	.21	.00	.20
Biotic	1758.2	Mussel biomass	30.21	0.16	<.0001	.03	.21
Abiotic	1687.7	Substrate (D_50_)	35.7	0.05	<.0001	.09	.27
		Velocity	18.84	1.85	<.0001		
		Depth	39.94	−1.24	<.0001		
Biotic and abiotic	1672.5	Mussel biomass	16.44	0.12	<.0001	.10	.26
		Substrate (D_50_)	27.54	0.04	<.0001		
		Velocity	14.61	1.63	<.0001		
		Depth	38.73	−1.27	<.0001		
*Corbicula* density							
Null	7468	–	–	3.09		.00	.34
Biotic	7357.3	Mussel biomass	46.057	0.25	<.0001	.026	.33
Abiotic	7429.4	Substrate (D_50_)	68.65	0.07	<.0001	.07	.37
		Velocity	7.59	1.44	.005		
		Depth	50.97	−1.91	<.0001		
Biotic and abiotic	7332.4	Mussel biomass	32.1	0.21	<.0001	.09	.36
		Substrate (D_50_)	56.69	0.07	<.0001		
		Velocity	4.67	1.13	.03		
		Depth	54.49	−1.96	<.0001		

Note

AIC values were used to compare hypothesis‐driven models to the null model. Variables included in the model had VIF < 5. Chi‐square values, model coefficients, and *p*‐values are shown for each variable. Marginal and conditional *R* ^2^ are given for each model.

### 3.4 Multivariate analysis of general patterns

**FIGURE 4:**
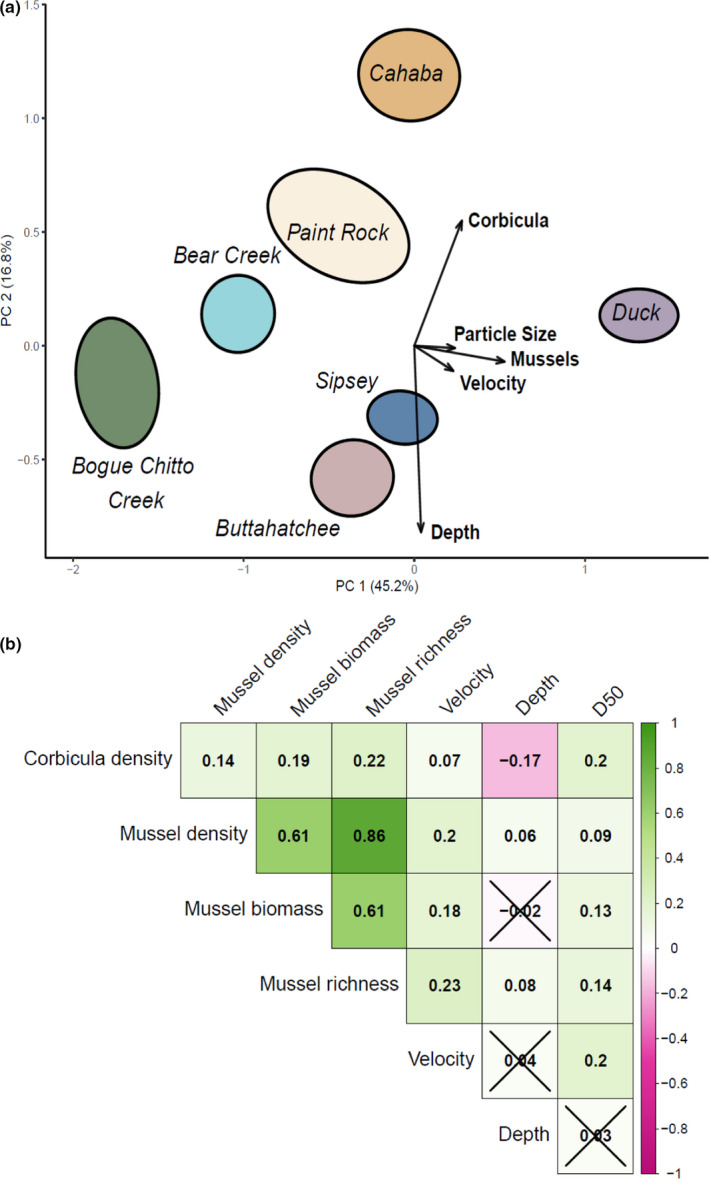
Ordination of principle components analysis of biotic and abiotic variables measured at the quadrat scale. River names are written in italics and correspond to 95% confidence ellipses for each river and vector arrows indicate latent biotic and abiotic gradients among rivers. “Corbicula” is Corbicula density (individuals m^−2^) and “Mussels” is mussel biomass (g m^−2^), richness, and density (individuals m^−2^). A single vector is shown for mussels because density and richness were strongly collinear (a). Plot of correlation matrix of variables included in quadrat scale generalized linear mixed models. Green indicates significant positive correlations, while pink indicated significant negative correlations. Boxes with an “X” are not statistically significant at *p* = .05 (b)

The first axis of the PCA explained 45.2% of the variation in biotic and abiotic factors measured at the quadrat scale, and the second axis explained 16.8% (Figure 4A). Ordination of the quadrat level factors were supported by PERMANOVA with centroids clearly separated (F_6,1445_ = 48.81, *p* = .001, *R* ^2^ = .17) and clear heterogeneity among rirvers (F_6,1445_ = 30.03, *p* = .001). PC1 was positively associated with the quadrat level mussel variables (richness, density, and biomass) and particle size and velocity. PC2 was positively associated with *Corbicula* density and negatively with water depth. Quadrats with more mussels, relatively larger substrates, and faster velocity fell out on the positive end of PC1 and were generally characteristic of quadrats sampled from the Duck River (95% confidence ellipse). Bogue Chitto Creek quadrats had the lowest density of mussels and were the most negative on the first axis. Quadrats from the Cahaba and Paint Rock River were the most positive on the second axis, where *Corbicula* densities were greatest, while the Sipsey and Buttahatchee Rivers both had the lowest densities of *Corbicula* and some of the deepest quadrats.

### 3.5 Correlation matrices at the patch‐ and reach‐scales

The correlation matrix of biotic and abiotic quadrat data showed that the mussel species richness, density, and biomass were positively correlated with each other, supporting the conclusion of the VIF analysis. *Corbicula* densities were positively correlated with mussel density (*r* = .14), biomass (*r* = .19), and richness (*r* = .22), suggesting strong overlap with mussels at the quadrat scale (Figure 4b). Velocity (*r* = .07) and substrate particle size (*r* = .20) were also positively correlated with *Corbicul*a density, but depth was negatively correlated (*r* = −17).

**FIGURE 5:**
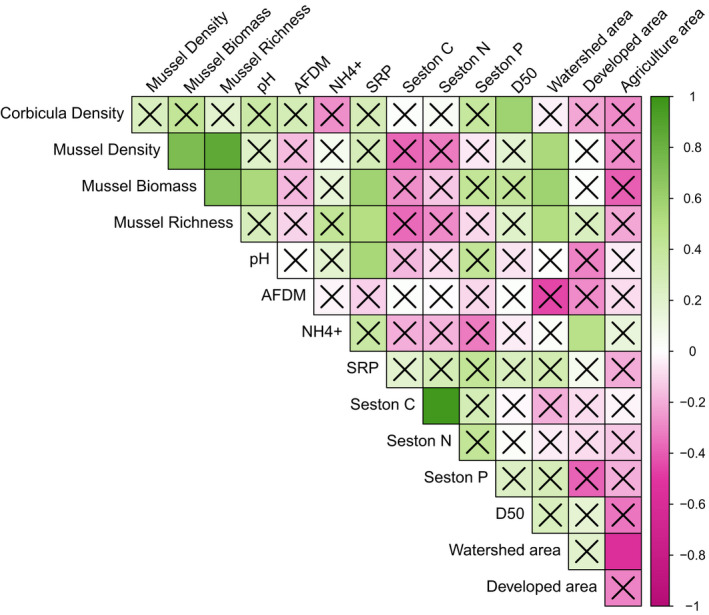
Correlation matrix of variables measured at mussel bed reaches. Green indicates significant positive correlations, while pink indicates significant negative correlations. Boxes with an “X” are not statistically significant at *p* = .05

The correlation matrix of mussel and habitat variables measured at the reach scale showed mean *Corbicula* density was positively correlated with D_50_ (Figure 5), suggesting it increases in reaches with larger particles sizes (*r* = .59). Mussel density (*r* = .54), biomass (*r* = .59), and richness (*r* = .51) were all positively correlated with watershed area. Mussel biomass (*r* = .58) was positively correlated with SRP. Mussel biomass (*r* = .55) and SRP (*r* = .59) were correlated with pH. Seston % C and % N were strongly and positively correlated with each other (*r* = .97). Water column ${\mathrm{NH}}_{4}^{+}$ was positively correlated with the proportion of developed landcover (*r* = .48). Larger watersheds were negatively related with the proportion of agricultural development (*r* = −.56).

## 4 DISCUSSION

In the rivers we studied, *Corbicula* was widespread, suggesting that its effects on the structure and functions of these ecosystems may be strong. Given the high density of *Corbicula* within mussel habitats and its functional similarity to native mussels, the potential for interactions with mussels is great. Studying the factors that influence the distribution of invasive species relative to native communities is important to understand the potential for positive and negative interactions (Ricciardi et al., 2020). We characterized factors associated with invasive *Corbicula* populations in seven environmentally heterogeneous rivers with diverse native mussel communities that have been differentially effected by anthropogenic pressures. We generated three hypothesis‐driven models to explain these occurrence and density patterns. Whereas all models were improvements over the null model, the best supported one included the full set of abiotic and biotic factors, suggesting that native mussels and *Corbicula* occupy similar stream habitats. This is contrary to our expectations that native mussels and *Corbicula* would limit each other's distribution as predicted by limiting similarity hypothesis (Macarthur & Levins, 1967) and previous works showing negative interactions (Ferreira‐Rodríguez, Fandiño, et al., 2018; Ferreira‐Rodríguez, Sousa, et al., 2018; Modesto et al., 2019, 2021; Vaughn & Spooner, 2006a). Thus, suggesting that niche requirements in these contemporary river habitats may not be limiting to either group or other mechanisms, such as positive interactions, may be at play (Silknetter et al., 2020).

Species interactions and physical habitat factors were important in explaining *Corbicula* occurrence and density in quadrats but varied strongly across the seven rivers even with our targeted sampling design within aggregations of mussels. Mussels and *Corbicula* share similar feeding and habitat requirements, therefore we expected competitive interactions to result in non‐overlapping distributions (Haag et al., 2020; Vaughn & Spooner, 2006a). Our analysis focused on mussel biomass because of the strong collinearity between species richness and density in the rivers we studied, and accounts for trade‐offs in space occupancy by many small‐bodied or few large‐bodied individuals. Using biomass as a metric to evaluate potential species interaction outcomes, we found only positive associations between mussels and *Corbicula* occurrence and density. Interestingly, most research addressing interactions between *Corbicula* and mussels has highlighted those with negative outcomes for mussels, but the strong positive association between each group in our study warrants investigation of positive interactions, such as the potential for mussels to facilitate *Corbicula* invasion. For example, *Corbicula* settlement and persistence within patches of mussels may be facilitated by the reduced turbulent shear stresses generated by high densities of mussels protruding from the sediment (Sansom et al., 2020). Nevertheless, facilitation of *Corbicula* still could result in harm to mussel populations through mechanisms other than competition. Indeed, recent efforts showed a negative relationship between survival of mussel larvae (glochidia) and *Corbicula* densities and hypothesized that the high filtration capacity of *Corbicula* may increase mortality of larval mussels by damaging larval shells when filtered by co‐occurring *Corbicula* or the high excretion capacity of *Corbicula* may lead to mortality of larval mussels by increasing local ammonia concentration (Modesto et al., 2019). The high potential for negative interaction outcomes for mussels highlights the need for further investigation into the potential for mussels to facilitate *Corbicula* invasions especially in regard to environmental context in which the interaction occurs (Ferreira‐Rodríguez et al., 2022), because the strength and direction (e.g., negative, neutral, positive) of the interactions may be context dependent (Albertson et al., 2021; Silknetter et al., 2020).

Ecological characteristics measured within quadrats reflected a suite of mussel assemblages and habitats occurring in the Mobile and Tennessee River basins that are determined by underlying physiography (Parmalee & Bogan, 1998; Williams et al., 2008). Benthic characteristics are an important factor in the distribution of benthic species, including mussels and *Corbicula* which live buried in benthic habitats and therefore may partition habitat at fine spatial scales. We hypothesized increasing probability of *Corbicula* occurrence and density in habitats where mussels already exist if habitat requirements are similar, but not limiting to either group. Our results show that larger substrate particle sizes (e.g., gravel and cobble), faster water velocity, and shallower depths positively influence *Corbicula* occurrence and density within mussel aggregations. *Corbicula* appears to be successful in similar habitats as native mussels within mussel beds suggesting a preference for the same habitat despite wide ecological gradients covered by our study. Whether *Corbicula* favor similar habitats outside of mussel bed reaches remains to be seen because our sampling sites may not represent the complete set of ecological conditions needed for maximum population growth. For example, most studies in North America of *Corbicula* habitat use are performed in sites with mussels (Ferreira‐Rodríguez, Sousa, et al., 2018; Vaughn & Spooner, 2006a). Although fine‐scale variables appear important to the distribution of *Corbicula* in these rivers, it could still be influenced by other factors not measured in this study. For example, another study of *Corbicula* habitat preference in the River Minho estuary in Spain showed a positive relationship with *Corbicula* biomass and the organic matter (OM) content of the sediment (Sousa et al., 2008), but did not mention the presence of mussels. Sediment organic matter produced by mussels via biodeposition may be an important factor influencing the distribution of *Corbicula*, because OM can be an alternative food source that is accessed through pedal feeding (Hakenkamp & Palmer, 1999; Hakenkamp et al., 2001) and may provide them with beneficial gut microbiota (Chiarello et al., 2022). It seems likely high densities of *Corbicula* may be supported in habitats with high organic matter content, such as mussel beds (Atkinson & Forshay, 2022; Vaughn & Hakenkamp, 2001). Thus, *Corbicula* invading into mussel bed habitats may not be limited by habitat parameters in these rivers and begs the question of whether OM biodeposition may facilitate *Corbicula* invasion into mussel beds.

Freshwater habitats of the Anthropocene are characterized by excessive nutrient loads due to difficulties managing non‐point source pollution inputs (e.g., fertilizer runoff) and may affect species interactions (Strayer, 2014). Studies of the trophic niche of *Corbicula* often conclude that it is highly flexible and overlaps with the trophic niche of native mussel species, but the extent varies with ecological context (Atkinson et al., 2010; Modesto et al., 2021), and such flexibility can facilitate invasion success (Moyle & Light, 1996; Olsson et al., 2009). We anticipated that nutrient loading would increase quality (increased nutrient content) and quantity of particulate food sources, alleviating competition for food resources between *Corbicula* and mussels, thereby allowing co‐occurrence. Whereas watersheds with more urban land cover did have increased water column ${\mathrm{NH}}_{4}^{+}$, our seston data did not indicate food resource quantity or quality was related to land use differences among watersheds. Moreover, bivalve variables were not correlated with nutrient or seston data. This supports the hypothesis that food quantity may not limit either *Corbicula* or mussel production in these rivers, and that *Corbicula* may be passengers of change in degraded mussel habitats (MacDougall & Turkington, 2005). Whether food quantity or quality limits mussel or *Corbicula* production in habitats where they co‐occur remains to be seen, particularly in low‐productivity habitats where mussel restoration efforts often focus (Strayer et al., 2019). Future efforts should systematically evaluate *Corbicula* population dynamics in habitats without mussels to separate the influence of mussels from nutrient context. Additionally, in situ or controlled lab experiments altering the seston quantity and C: nutrient ratios could be used to identify food threshold elemental ratios (Frost et al., 2006) that optimize *Corbicula* or mussel species growth.

The unexpected positive association between *Corbicula* and mussel distributions represents a snapshot in time that may not reflect the temporal variability of *Corbicula* and mussel population dynamics, which unfold at different time scales, which is needed to understand their direct or indirect interactions. For example, *Corbicula* is quite vulnerable to high temperatures and low dissolved oxygen which can lead to mass mortality events resulting in water quality issues that can harm mussels (McDowell et al., 2017; McDowell & Sousa, 2019). Yet, *Corbicula* can recover quickly (within a year) from such disturbances, while mussel populations take decades to recover from disturbances due to their slow maturation (Haag, 2012). Future efforts that combine life history traits and population estimates could be used to assess mussel and *Corbicula* population responses to disturbances related to global change. Further experimental work is warranted to disentangle interactions between *Corbicula* and mussels to address how their interactions change across environmental gradients.

## 5 CONCLUSION

Disentangling the factors that control invasive species’ distribution and abundance is challenging, especially once a species is established. Our data across two spatial scales indicated high spatial overlap when considering the occurrence of an invasive species in targeted native communities and highlights support for our alternative hypotheses of non‐limiting resources (i.e., low competition), or that *Corbicula* may be passengers of change in degraded rivers. Additionally, our study brings to light the hypothesis of facilitation of *Corbicula* into mussel beds via mussel activities, but the underlying mechanisms are unknown or speculative and warrant further investigation. When invasive species co‐occur with functionally similar species, the potential competitive outcome may be especially harmful to the native fauna because competitive interactions should be strong. Large‐scale and long‐term monitoring programs in place for imperiled species, such as native mussels, should incorporate systematic sampling of functionally similar invasive species, such as *Corbicula*, to provide data on range overlaps, population growth trajectories, potential interactions with native communities, and altered ecosystem function, and to inform future management and conservation strategies.

## CONFLICT OF INTEREST

None declared.

## AUTHOR CONTRIBUTIONS

**Taylor E. Kelley:** Conceptualization (supporting); Data curation (supporting); Formal analysis (supporting); Investigation (equal); Methodology (supporting); Visualization (supporting); Writing – original draft (equal); Writing – review & editing (lead). **Garrett W. Hopper:** Conceptualization (equal); Data curation (lead); Formal analysis (lead); Investigation (equal); Methodology (supporting); Visualization (lead); Writing – original draft (equal); Writing – review & editing (supporting). **Irene Sánchez González:** Conceptualization (supporting); Investigation (supporting); Methodology (supporting); Visualization (supporting); Writing – original draft (supporting); Writing – review & editing (supporting). **Jamie R. Bucholz:** Conceptualization (supporting); Investigation (supporting); Methodology (supporting); Writing – original draft (supporting); Writing – review & editing (supporting). **Carla L. Atkinson:** Conceptualization (equal); Funding acquisition (lead); Investigation (supporting); Methodology (supporting); Resources (supporting); Supervision (supporting); Writing – original draft (supporting); Writing – review & editing (supporting).

## Supporting information

Supplementary Material

## Data Availability

Data supporting the results of this paper can be found at the Open Science Framework https://doi.org/10.17605/OSF.IO/M2U9J.
