# Supplementary material for: Identifying potential drivers of distribution patterns of invasive Corbicula fluminea relative to native freshwater mussels (Unionidae) across spatial scales
Source: Ecol Evol. 2022 Mar 18;12(3):e8737. doi: 10.1002/ece3.8737 (PMC8933331; doi:10.1002/ece3.8737)
Supplement: Supplementary file 1 — Supplementary Material [file ECE3-12-e8737-s001.docx]

**Appendix: Identifying drivers of distribution patterns of invasive *Corbicula fluminea* and native freshwater mussels (Unionidae) across spatial scales**

Table S1. Biotic characteristics measured at 20 mussel bed reaches in the Mobile ^(M)^ and Tennessee ^(T)^ River Basins during 2019 and 2020.

| Site | *Corbicula* density (ind. m^-2^) | Mussel density (ind. m^-2^) | Mussel biomass (g STDM m^-2^) | Mussel richness | |
| --- | --- | --- | --- | --- | --- |
| Bear 1 ^T^ | 3.58 | 2.93 | 3.60 | 11 |  |
| Bear 2 | 2.42 | 1.49 | 2.40 | 11 |  |
| Bogue 1^M^ | 2.05 | 2.26 | 3.14 | 12 |  |
| Bogue 2 | 6.40 | 0.60 | 0.46 | 4 |  |
| Buttahatchee 1^T^ | 8.41 | 4.37 | 2.18 | 13 |  |
| Buttahatchee 2 | 1.70 | 5.32 | 4.80 | 15 |  |
| Cahaba 1^M^ | 73.29 | 4.74 | 16.44 | 12 |  |
| Cahaba 2 | 107.20 | 4.92 | 16.09 | 10 |  |
| Cahaba 3 | 6.73 | 4.87 | 9.67 | 7 |  |
| Cahaba 4 | 134.60 | 2.80 | 8.21 | 9 |  |
| Duck 1^T^ | 61.43 | 18.14 | 40.24 | 32 |  |
| Duck 2 | 22.40 | 13.60 | 15.12 | 25 |  |
| Duck 3 | 92.18 | 18.92 | 21.48 | 21 |  |
| Duck 4 | 4.46 | 4.92 | 18.64 | 15 |  |
| Paint Rock 3^T^ | 36.92 | 4.50 | 1.90 | 15 |  |
| Sipsey 1^M^ | 4.67 | 0.55 | 0.97 | 8 |  |
| Sipsey 2 | 4.76 | 11.40 | 11.43 | 15 |  |
| Sipsey 3 | 2.26 | 11.96 | 7.37 | 18 |  |
| Sipsey 4 | 16.20 | 23.86 | 10.73 | 23 |  |
| Sipsey 5 | 2.39 | 9.07 | 4.12 | 17 |  |

Table S2. Abiotic characteristics measured at 20 mussel bed reaches during 2019 and 2020.

| Site | Watershed area (km^2^) | Agricultural area (km^2^) | Developed area (km^2^) | D_50_ | pH | NH_4_^+^ (µg/L) | SRP (µg/L) | AFDM (mg/L) | Seston C% | Seston N% | Seston P% |
| --- | --- | --- | --- | --- | --- | --- | --- | --- | --- | --- | --- |
| Bear 1 | 1729.07 | 349.65 | 116.16 | 16 | 6.98 | 16.43 | 9.17 | 2.74 | 3.88 | 0.52 | 0.20 |
| Bear 2 | 1869.50 | 792.84 | 69.64 | 8 | 6.85 | 14.31 | 10.12 | 2.97 | 2.93 | 0.40 | 0.33 |
| Bogue 1 | 729.36 | 333.04 | 71.67 | 11 | 7.35 | 18.34 | 59.29 | 7.00 | 4.79 | 0.62 | 0.19 |
| Bogue 2 | 844.91 | 88.10 | 49.04 | 11 | 7.24 | 11.36 | 19.08 | 6.60 | 10.01 | 1.32 | 0.32 |
| Buttahatchee 1 | 1999.85 | 147.86 | 476.91 | 16 | 6.08 | 17.56 | 6.82 | 4.09 | 3.45 | 0.41 | 0.08 |
| Buttahatchee 2 | 2111.51 | 101.32 | 696.73 | 11 | 6.95 | 21.63 | 9.93 | 4.74 | 2.87 | 0.39 | 0.13 |
| Cahaba 1 | 564.87 | 269.96 | 59.70 | 16 | - | 5.73 | 13.07 | - | 3.78 | 0.41 | - |
| Cahaba 2 | 1134.10 | 160.48 | 29.23 | 16 | 7.61 | 10.78 | 6.97 | 13.73 | 3.20 | 0.44 | 0.90 |
| Cahaba 3 | 2438.06 | 177.66 | 116.72 | 11 | 7.68 | 9.70 | 7.24 | 1.53 | 2.52 | 0.36 | 0.32 |
| Cahaba 4 | 2702.09 | 204.30 | 147.09 | 16 | 7.44 | 7.23 | 10.77 | 3.98 | 3.15 | 0.29 | - |
| Duck 1 | 2368.48 | 172.55 | 657.25 | 16 | 7.93 | 26.97 | 105.80 | 3.48 | 2.45 | 0.36 | 0.20 |
| Duck 2 | 2440.49 | 178.07 | 120.53 | 11 | 7.96 | 17.36 | 112.29 | 4.40 | 3.29 | 0.48 | 0.32 |
| Duck 3 | 2632.58 | 192.84 | 132.68 | 16 | 7.79 | 14.64 | 95.12 | 3.97 | 2.33 | 0.30 | 0.49 |
| Duck 4 | 3119.36 | 287.33 | 185.59 | 11 | 7.86 | 24.27 | 104.29 | 4.84 | 5.86 | 0.96 | 0.91 |
| Paint Rock 3 | 1055.86 | 164.07 | 27.84 | 11 | 7.47 | 17.29 | 7.04 | 9.97 | 2.46 | 0.32 | 0.10 |
| Sipsey 1 | 673.14 | 321.91 | 70.71 | 8 | 7.96 | 28.41 | 9.55 | 2.96 | 2.19 | 0.24 | 0.09 |
| Sipsey 2 | 1258.75 | 121.26 | 22.71 | - | 8.19 | 16.21 | 11.25 | 4.77 | 2.83 | 0.36 | 0.11 |
| Sipsey 3 | 1674.36 | 358.39 | 118.80 | 8 | 7.30 | 15.39 | 6.68 | 3.37 | 3.21 | 0.41 | 0.35 |
| Sipsey 4 | 1746.02 | 737.32 | 65.92 | 11 | 7.17 | 16.35 | 7.46 | 1.67 | 3.21 | 0.38 | 0.07 |
| Sipsey 5 | 1882.03 | 133.14 | 416.62 | 8 | 7.13 | 14.96 | 7.69 | 1.80 | 3.21 | 0.44 | 0.09 |
